# A Highly Sensitive Method for the Detection of Hydrolyzed Gluten in Beer Samples Using LFIA

**DOI:** 10.3390/foods12010160

**Published:** 2022-12-28

**Authors:** Verónica Segura, Miguel Ángel Siglez, Ángela Ruiz-Carnicer, Izaskun Martín-Cabrejas, María van der Hofstadt, Encarnación Mellado, Isabel Comino, Carolina Sousa

**Affiliations:** 1 Department of Microbiology and Parasitology, Faculty of Pharmacy, University of Seville, 41012 Seville, Spain; 2 Biomedal S.L., 41900 Camas, Spain; 3 Food Technology Department, Veterinary Faculty, Complutense University of Madrid, 28040 Madrid, Spain; 4 ALINUA, Food and Nutrition Cabinet Health Science Faculty, University of Alicante, UA, 03690 Alicante, Spain

**Keywords:** celiac disease, hydrolyzed peptides, gluten immunogenic peptides

## Abstract

Most gluten analysis methods have been developed to detect intact gluten, but they have shown limitations in certain foods and beverages in which gluten proteins are hydrolyzed. Methods based on G12/A1 moAbs detect the sequences of gluten immunogenic peptides (GIP), which are the main contributors to the immune response of celiac disease (CD). Immunogenic sequences with tandem epitopes for G12/A1 have been found in beers with <20 mg/kg gluten, which could be consumed by CD patients according to the Codex Alimentarius. Therefore, an accurate method for the estimation of the immunogenicity of a beer is to use two moAbs that can recognize celiac T cell epitopes comprising most of the immunogenic response. Here, a specific and sensitive method based on G12/A1 LFIA was developed to detect GIP in beers labeled gluten-free or with low gluten content, with an LOD of 0.5 mg/kg. A total of 107 beers were analyzed, of those 6.5% showed levels higher than 20 mg/kg gluten and 29% showed levels above the LOD. In addition, G12/A1 LFIA detected gluten in 15 more beer samples than competitive ELISA with another antibody. Despite their labeling, these beers contained GIP which may cause symptoms and/or intestinal damage in CD patients.

## 1. Introduction

Gluten refers to a complex set of storage proteins present in the endosperm of cereals, such as wheat, rye, barley, oat, and their derivatives. It comprises two protein fractions: prolamins (monomeric and alcohol-soluble proteins) and glutelins (polymeric and alcohol-insoluble proteins) [1,2]. Prolamins and glutelins are responsible for the functional properties of cereal dough and are characterized by a high content of proline and which makes them resistant to gastrointestinal digestion and encouraging their deamidation by tissue transglutaminase. Consequently, the consumption of these proteins causes adverse reactions in people suffering from gluten-related disorders (GRDs), such as wheat allergy, non-celiac gluten sensitivity (NCGS), dermatitis herpetiformis, gluten ataxia, and celiac disease (CD) [3,4,5,6].

Among GRDs, CD has been extensively studied, and the role of gluten in its pathogenesis has been clearly identified. It is a systemic process that produces chronic enteropathy of the small intestine, and it develops through an inadequate immune response to gluten in genetically predisposed individuals [7,8,9,10]. Currently, the only effective treatment is a gluten-free diet (GFD) that relies on the consumption of naturally gluten-free foods, such as animal-based products, fruits, vegetables, legumes, and nuts, as well as gluten-free dietary products that do not contain more than 20 mg/kg of gluten according to the Codex Alimentarius [11], including beer and certain other beverages [12,13,14].

Beer is a popular alcoholic beverage that has been consumed worldwide for millennia [15]. It is traditionally composed of four ingredients: malted barley, water, hops, and yeast. Currently, to meet consumer tastes and needs, new types of beers have become prominent, including nonalcoholic, fruit-flavored, craft, and gluten-free [16]. However, for patients with CD, the safety of beers derived from gluten-containing grains, particularly wheat and barley, remains a considerable concern [17]. The amount of gluten produced depends on the various stages of the manufacturing process. During mashing and fermentation, most of the protein fraction is removed [18,19,20,21], resulting in short peptide fragments and free amino acids remaining in the beer [22,23]. However, precipitation, lautering, and enzymatic hydrolysis do not abolish the gluten epitopes known to trigger CD; consequently, several studies have reported adverse responses of patients with CD to commercial beers. Indeed, the potential diversity in the generation of sequences, relative abundance, and the extent of the resulting gluten peptides are almost unlimited [24,25].

Enzyme-linked immunosorbent assay (ELISA) is a standard method for the detection and quantification of gluten in food. However, the ability of various ELISA kits to determine the true gluten content is questionable [26,27], as currently available methods have been developed to detect intact gluten but show limitations when quantifying hydrolyzed gluten [14]. Poor recognition of some of major immunodominant gluten peptides can result in an underestimation of the immunogenicity of some foods, especially hydrolysates, in which these peptides are more readily bioavailable to trigger the disease to a much greater extent than undigested gluten. This uncertainty arises when interpreting ELISA results for the quantification of hydrolyzed gluten in terms of equivalent amounts of intact gluten [28,29,30]. Therefore, the underestimation or even lack of detection of true immunogenicity involves a significant risk for patients with CD.

Immunological methods (ELISAs and lateral flow immunochromatographic assays (LFIAs)) have been developed based on G12 and A1 monoclonal antibodies (moAbs). These moAbs can detect gluten immunogenic peptides (GIP), which are gluten fragments resistant to gastrointestinal digestion and the main factors responsible for the immune response of patients with CD [31]. Based on these moAbs, immunogenic peptides have been found in beers with a gluten content of < 20 mg/kg, which, according to the Codex Alimentarius regulations, could be consumed by patients with CD [23,28,29,32,33,34,35,36,37]. Further analysis of these beers revealed that the reactive fractions of G12/A1 moAbs displayed GIP sequences using matrix-assisted laser desorption/ionization time-of-flight (MALDI TOF/TOF), and most of the immunogenic sequences identified showed tandem epitopes for G12 and A1 moAbs [32]. Therefore, the best way to measure the level of immunogenicity of a beer is to use two moAbs that can detect gluten peptides involved in most of the immunogenic response to these proteins. These moAbs must recognize sequences that do not overlap each other.

Recent advances in food allergen analysis have emphasized the need for rapid immunoanalytical methods such as LFIAs. These tests are currently the most desired methods, as they offer a low-cost alternative to conventional laboratory tests [38]. This type of test is a suitable, fast, and easy-to-use option for the analysis of mycotoxins and allergens. Currently, the main application of LFIAs in allergen management is the detection of trace amounts of allergens on surfaces. However, they are also suitable for the detection of trace amounts of allergens in food, as they are faster and easier methods to perform than ELISAs. Additionally, some LFIAs can detect proteins not only in raw products, but also in processed food products. They are commercially available for various analytes (gluten, peanut, hazelnut, soy, milk, fish, mustard, crustacea, peanut, etc.), and have been validated for numerous matrices [38,39,40,41,42].

This study aimed to develop a specific and sensitive method (developed following the Quantifying Uncertainty in Analytical Measurement issued by Eurachem in 1995 and its revised version issued jointly with CITAC in 2020 [43]) based on G12/A1 LFIA, capable of rapidly and reliably detecting gluten present in beer samples labeled as gluten-free without the need for an extraction process, and with a limit of detection (LOD) of 0.5 mg/kg.

## 2. Materials and Methods

### 2.1. Reference Standards

33-mer peptide (LQLQPFPQPQLPYPQPQLPYPQPQLPYPQPQPF), Prolamin Working Group (PWG)-gliadin and hydrolyzed wheat prolamin of the wheat variety CUBUS (PTW-Cubus; aromaLAB, Planegg, Germany) were used as reference standards.

### 2.2. Beer Samples

A total of 107 commercial beers labeled gluten-free or low-gluten were obtained, which included some craft beers from different countries of Europe (Belgium, Czech Republic, France, Finland, Germany, Italy, Luxembourg, Poland, Portugal, Spain, and Switzerland) as well as other parts of the world. Descriptive factors such as labeling, yeast style, and wheat inclusion as raw materials were analyzed. Additionally, two high-gluten wheat commercial beers (Germany) were also acquired for use as a positive gluten control.

### 2.3. Spiked Samples

Different beer samples with gluten concentrations below the LOD were spiked with PTW-Cubus (0.5 mg/kg PTW-Cubus) to investigate a possible matrix effect and evaluate the recovery.

### 2.4. Techniques Employed

#### 2.4.1. Development of a New Method

##### Lateral Flow Immunochromatographic Assays (LFIAs)

A G12/A1 moAbs strip was used to develop an analytical method based on the reaction of gluten peptides present in beer samples with colored G12 and A1 moAbs already fixed in the absorption zone. Gluten peptides present in beer samples were bound to the antibody labeled with the detection signal during membrane migration to form a labeled antigen–antibody complex. This complex flowed along the membrane to form a sandwich-like immune complex with the captured moAb immobilized on the test line via the free epitope of the analyte. The result was positive, if a red/pink line appeared in the result zone of the membrane, providing a signal. The absence of this line and therefore a signal below the established limit suggested a negative result. The blue control line was always used as a test control.

##### Analysis of Samples and Data

**Figure 1:**
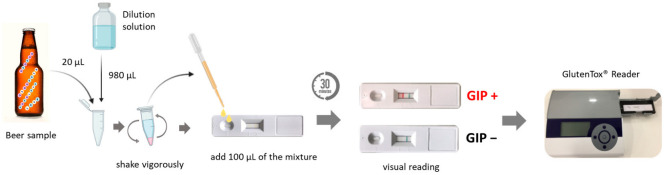
Working procedure for gluten analysis in beer samples. GIP, gluten immunogenic peptides; LFIA, lateral flow immunochromatographic assays.

The beer samples and reagents were kept at room temperature (15–25 °C). The samples were then diluted at 1:50, using 20 µL of the beer sample and 980 µL of the dilution solution. Subsequently, 100 µL of the diluted sample were added to the shells of the strips and incubated for 30 min. Finally, the strips were ready for visual analysis. The peak area of the immunochromatographic strip was determined using a GlutenTox^®^ Reader (Hygiena, Seville, Spain) (Figure 1).

GlutenTox^®^ Reader (Hygiena, Seville, Spain) integrates electronics and LFIA Studio software (Hygiena, Seville, Spain) for data processing [44]. The peak area values for each sample and the control were obtained using this software. Spreadsheets were used to compile raw data, and the results were considered positive when the reading of the test line was above the negative sample value plus three times the standard deviation. If this criterion was not met, the test would yield a negative result. The values obtained using the G12/A1 LFIA performed in duplicate for each sample. The results were semi-quantitative and expressed in the unit of mg/kg according to the minimum quantification dilution detected.

#### 2.4.2. Enzyme-Linked Immunosorbent Assay (ELISA)

Gluten content in beer was determined using the ELISA RIDASCREEN^®^ Gliadin competitive R5 moAb (R7021, R-Biopharm, Darmstadt, Germany). All the samples were analyzed in duplicates according to the manufacturer’s instructions. The obtained data were expressed as the mean and standard deviation.

### 2.5. Statistical Anlysis

Relative affinity curves were obtained by plotting the percent maximum absorbance against the reference standard concentration (ng/mL). The Sigma Plot, version 12.0, Systat Software, Inc., software package (San José, CA, USA) was used to calculate the IC_50_ and cross-reactivity (CR) for each standard. The IC_50_ was defined as the concentration of the line that reduced the peak absorbance by 50% in the assay. Cross-reactivity (CR) was calculated as (IC_50_ of the standard with the highest affinity for the antibody/IC_50_ of each tested standard) × 100.

## 3. Results and Discussion

### 3.1. Validation of the New Analytical Method

The proposed method was evaluated according to the Eurachem guidelines based on studies of sensitivity, specificity, suitability, precision, and robustness [43]. Similar tests have already been validated for the detection of gluten in food and beverages (with non-hydrolyzed gluten) with different compositions and levels of processing, from raw materials to processed food [39]. In this study, the method was validated for beer hydrolysates.

#### 3.1.1. Immunochromatographic Strip Sensitivity and the LOD

The sensitivity was determined based on the characteristics of the immunochromatographic strips and the batch-to-batch effect. It was calculated using spikes from 0 to 25 ng/mL PTW-Cubus in a dilution solution with visual measurements after 30 min. These assays were performed in triplicate (analysis I–II–III). All three assays with spikes of 5–25 ng/mL of PTW-Cubus were visually positive; however, only one of the three assays showed positive results with spikes of 2.5 ng/mL PTW-Cubus, and all the assays showed negative results when containing <2.5 ng/mL PTW-Cubus (Table S1).

Based on the sensitivity of PTW-Cubus, the LOD of the method was determined as gluten (mg/kg) = [S × FE × DF × 2]/1000, where S is the calculated sensitivity; DF is the dilution factor; FE is the extraction factor equaling to 1; gliadin-to-gluten ratio is 2. A sensitivity of 5 ng/mL PTW-Cubus was established for reading at 30 min and test dilution of 1:50, resulting in an LOD of 0.5 mg/kg for gluten.

#### 3.1.2. Strip Reader Detection Capacity

To determine the detection capacity of the reader used in the test, the measurements obtained in the analysis of the beer samples (<LOD) were compared with the measurements of the same beer samples spiked with 0.5 mg/kg PTW-Cubus.

First, the average peak area value offered by the reader was established, when five beer samples with the gluten content lower than the LOD were analyzed five times each. In most of the validation protocols reported in the literature, the verification of the method [45] was calculated by executing at least five determinations per concentration and a deviation below 15% or 20% of the expected value [46] as the acceptance criterion (excluding negative samples) [47]. The results showed a negative visual interpretation in all strips. However, the reader detected a signal in 8 of the 25 samples analyzed, with a mean peak area of 35.1 ± 9.7 (Table S2). Based on these results, a minimum peak area of approximately 70 was established to consider a sample as positive. This was calculated by adding three times the deviation to the value of the mean peak area (35.1) [43,48].

Furthermore, the validity of this gluten-detection method was determined using 0.5 mg/kg of PTW-Cubus spiked in four samples (<LOD) five times each. All the strips were positive on visual interpretation, and the results provided by the strip reader established a mean peak area of 256.8 ± 26.5 in these samples (Table 1).

**Figure 2:**
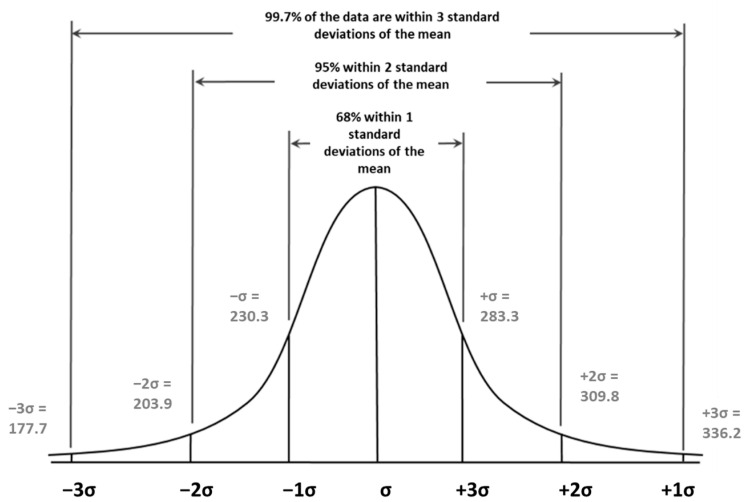
The normal distribution and range of variation in beer samples (gluten < LOD) spiked with 0.5 mg/kg PTW-Cubus. σ, coefficient of variation.

Once the average results of the peak area and standard deviations were obtained using the strip reader, the range of reading variation was calculated as a function of the coefficient of variation (σ). Furthermore, confidence intervals around the mean were determined, with confidence levels of 99.7% (peak area: 283.3–230.3), 95% (peak area: 309.8–203.9), and 68% (peak area: 336.2–177.7). Finally, the range ± 2σ was established as the criterion for considering a result as valid, since a 95% coverage was observed in that range (Figure 2) [49].

#### 3.1.3. Accuracy and Precision

According to the Eurachem guide, accuracy and precision indicate the ability of the method to obtain results within the established range of interest. According to these guidelines, the results must be obtained from an average of 6 to 15 replicates of each material, with the same equipment, analyst, and laboratory and in a short period [43].

In this study, accuracy was calculated by taking 18 measurements of 2 beer samples (<LOD) spiked with 0.5 mg/kg PTW-Cubus. These 18 measurements were performed on the same day and from 3 different extractions, each analyzed in triplicates (Table 2). Meanwhile, precision was achieved by performing 36 measurements of 2 beer samples (<LOD) spiked with 0.5 mg/kg PTW-Cubus. These 36 measurements were carried out on 2 different days, and each measurement was analyzed in triplicates.

All the strips were positive on visual interpretation. Of the 18 replicates analyzed to calculate the accuracy, 16 were within the range (peak area: 203.9–309.8), indicating an accuracy of 88.9% on day 1 and 100% on day 2. In addition, 34 of the 36 replicates used to calculate the precision were within the established range (peak area: 203.9–309.8), indicating a precision of 94.5% (Table 2).

### 3.2. Determination of the Relative Affinity from Different Reference Standards

To confirm the detection capacity of G12/A1 LFIA against the most widely used reference standards in immunological assays, we tested 33-mer peptide, PWG-gliadin, and PTW-Cubus (hydrolyzed wheat prolamin). The affinity of moAbs for the antigens was quantified by calculating the concentration of the antigen, resulting in a 50% reduction in the peak signal in the LFIA (IC_50_) and CR. The results were compared with those obtained from R5 competitive ELISA.

**Figure 3:**
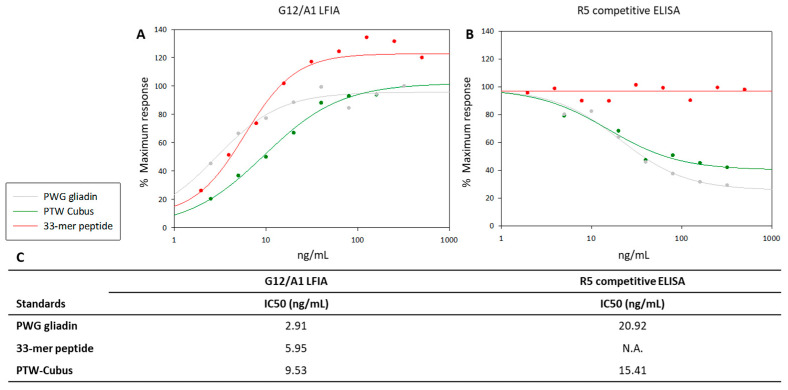
Relative affinity of G12/A1 LFIA and R5 competitive ELISA for different reference standards. (**A**) G12/A1 LFIA. (**B**) R5 competitive ELISA. (**C**) Standard reference curves. IC_50_ and CR were obtained for G12/A1 LFIA and R5 competitive ELISA. The IC_50_ is defined as the concentration of the line that reduces the peak absorbance by 50% in the assay. These assays were performed in duplicates. In the figure the gray points correspond to PWG-gliadin; the green points, PTW-Cubus; and the red dots, 33-mer peptide. LFIA, lateral flow immunochromatographic assays; N.A., not applicable.

PWG-gliadin showed the highest reactivity with G12/A1 moAbs (IC_50_, 2.91; CR, 100%). In addition, the 33-mer peptide (IC_50_, 5.95; CR, 48.91%) and gliadin PTW-Cubus (IC_50_, 59.53; CR, 30.54%) were also detected with high sensitivity using these moAbs (Figure 3A,C).

The results obtained using the R5 competitive ELISA demonstrated that this antibody was able to detect PWG-gliadin and gliadin PTW-Cubus, although with lower affinity than G12/A1 moAb, and the 33-mer peptide was not detected using moAb R5 (Figure 3B). Different research groups have shown that the α-gliadin 33-mer is poorly identified with R5 competitive ELISA [23,50,51]. By contrast, analysis of T-cell reactivity and enzyme protein detoxification showed that the signals of G12 and A1 moAbs correlate with the potential toxicity of the sample for patients with CD [23,32,52,53,54,55]. The recognition profiles of G12 and A1 moAbs have been extensively characterized [23,52,53]. Moreno et al. [56] showed that most GIP (responsible for 80–95% of celiac T-cell immunoreactivity) react with G12 moAb.

### 3.3. Gluten Analysis in Beer

Gluten analysis was carried out in 107 beers, with 88 labeled gluten-free, 12 low in gluten or with traces of gluten (non-specific labeled), and 7 not labeled for gluten or artisanal. Additionally, we tested two high-gluten beer samples as positive controls. The beer samples were grouped according to their cereal content in their labeling; based on this classification, 65% (70/107) contained cereals with gluten, 16.8% (18/107) contained a mixture of cereals with and without gluten, and only 4.7% (5/107) of the beer samples contained exclusively gluten-free cereals (maize, millet, buckwheat, and rice). According to the type of yeast used in the preparation, 56.8% (49/107) were made with lager yeast, and 43% (46/107) were prepared with ale yeast (Table 3).

**Figure 4:**
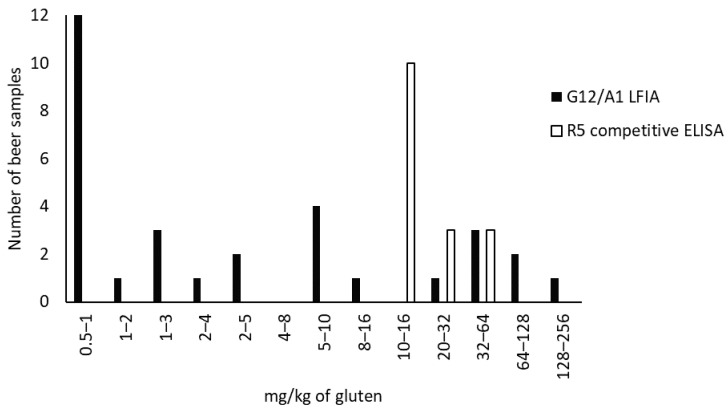
Number of beers in each gluten mg/kg range resulting from the G12/A1 LFIA or R5 competitive ELISA. ELISA, enzyme-linked immunosorbent assay; LFIA, lateral flow immunochromatographic assays; ppm, parts per million.

The gluten content was below the LOD (0.5 mg/kg gluten) in 71% (76/107) of the samples analyzed using G12/A1 LFIA. Our results showed that 29% (31/107) of the analyzed samples were positive in the range of 0.5–256 mg/kg of gluten (Figure 4), although they were labeled as gluten-free or low-gluten. In addition, 6.5% (7/107) contained levels higher than 20 mg/kg of gluten. This could be related to the brewing process, ingredients, the yeast used, and other factors. The addition or absence of malted cereal and the use of clarification processes, such as filtration, centrifugation, or the cold step, can influence the gluten content in beer [19]. Therefore, the inclusion of additional steps in the production process and/or the use of enzymes to hydrolyze gluten (e.g., PEP) could modify the amount of gluten present [34]. It should be noted that 74.2% (23/31) of the analyzed beer samples contained gluten-containing cereals on their labels. Furthermore, the number of beers with a positive result for gluten and ale yeast was higher than the number of those containing lager yeast (51.6% (16/31) and 32.3% (10/31), respectively). The influence of yeast fermentation on gluten content in the final product was previously noted by Tanner et al. [57] and Fernandez-Gil et al. [30], who highlighted the importance of selecting lager yeasts or excluding cereals with gluten in the brewing process to reduce the gluten content of the final product.

Additionally, the results obtained with G12/A1 LFIA were compared with those of R5 competitive ELISA, which revealed that 85% (91/107) of the samples were below the limit of quantification (LOQ) (<10 mg/kg of gluten) and 15% (16/107) showed positive results ranging from 10.8 to 63.1 mg/kg of gluten (Figure 4). This was a predictable result, as G12/A1 LFIA had a lower LOD than R5 competitive ELISA. Therefore, G12/A1 LFIA detected gluten in 15 more beer samples (beers Q to EE), 73% (11/15) of which contained gluten-containing cereals on their labels.

Notably, nine beers showed values from 10 to 16 mg/kg gluten using R5 competitive ELISA, four of which had a gluten content ranging from 20 to 128 mg/kg according to G12/A1 LFIA; therefore, these beers should not be consumed by patients with CD. Several studies have indicated that R5-based methods can overestimate or underestimate the net immunotoxicity of beer for patients with CD [56,58,59]. Moreover, in August 2020, the US FDA issued a dossier (FDA-2014-N-1021-0560), stating that it does not know “any scientifically valid analytical method that is effective in accurately detecting and quantifying the protein content of gluten in fermented or hydrolyzed foods in terms of equivalent amounts of intact gluten proteins”, this includes R5 competitive ELISA. Additionally, this release emphasized the lack of detection of gluten using R5 competitive ELISA, this antibody does not identify all immunogenic amino acid sequences and gluten may be present in a form not detectable with R5 competitive ELISA [60]. Furthermore, a claim stated in technical and marketing documents of R5 immunomethods is their reactivity to the 33-mer α-gliadin. However, in this study, the results revealed that R5 did not react with this peptide (Figure 3).

Several patients with CD experience adverse symptoms after ingesting gluten-free labeled beers, possibly because gluten proteins in beer are hydrolyzed, making GIP more readily bioavailable to trigger the disease to a much greater extent than undigested gluten [24]. These traces of gluten may not be detected using certain analytical methods, but they pose a risk to patients with CD because they preserve their toxicity. If a sufficient amount is ingested, which is likely to be in a drink, it can induce an immune response in the intestinal epithelium of celiac patients. Therefore, it is necessary to review the current gluten-free legislation of the European Union (EU), published in 2009 and regulated in 2012, which specifies two subgroups: gluten-free (≤20 mg/kg) and low-gluten content (21–100 mg/kg) [61]. In addition, lower local limits of gluten have already been established in many countries, ranging from 20 mg/kg in Spain, Italy, the UK [11], Canada, and the USA to 10 mg/kg of gluten in Argentina and 3 mg/kg of gluten in Australia, New Zealand, and Chile [62,63]. Consequently, this more sensitive method was developed to detect gluten concentrations of up to 0.5 mg/kg in beer, so that it can be safely consumed by patients with CD without causing symptoms or intestinal damage.

## 4. Conclusions

In this study, a highly sensitive and easy-to-use method based on LFIA was developed that requires no extraction and has an LOD of 0.5 mg/kg of gluten. Furthermore, it can measure the level of bioavailable GIP in hydrolyzed foods. Our results demonstrated that, although different regulations allow beer products to contain less than 20 mg/kg of gluten to be considered gluten-free, they still contain GIP, which should not be consumed by patients with CD. Consequently, we recommend reducing the limit of gluten-free labeling of beers according to the availability of sensitive methods, such as the one described in this study, that can detect bioavailable gluten.

## Figures and Tables

**Table 1:** Peak area results of beer samples (gluten < LOD) spiked with 0.5 mg/kg PTW-Cubus, obtained by the strip reader.

Beer Samples	Spiked Sample	Peak Area
Beer I	1	282.0
	2	278.5
	3	270.2
	4	227.0
	5	274.6
Beer II	6	250.6
	7	251.7
	8	263.6
	9	273.9
	10	309.0
Beer III	11	288.0
	12	232.3
	13	279.6
	14	214.3
	15	228.1
Beer IV	16	233.3
	17	279.2
	18	244.8
	19	234.8
	20	220.8

**Table 2:** Determination of accuracy and precision in beer samples (gluten < LOD) spiked with 0.5 mg/kg PTW-Cubus.

Day	Beer	Extraction	Analysis	Peak Area	Inside the Range	Accuracy (%)	Precision (%)
Day 1	Beer I	Beer Ia	Beer Ia	277.5	yes	88.9%	94.5%
			Beer Ia	305.1	yes		
			Beer Ia	290.4	yes		
		Beer Ib	Beer Ib	196.9	no		
			Beer Ib	278.1	yes		
			Beer Ib	267.3	yes		
		Beer Ic	Beer Ic	186.9	no		
			Beer Ic	279.0	yes		
			Beer Ic	217.9	yes		
	Beer II	Beer IIa	Beer IIa	255.9	yes		
			Beer IIa	259.2	yes		
			Beer IIa	268.9	yes		
		Beer IIb	Beer IIb	248.2	yes		
			Beer IIb	297.3	yes		
			Beer IIb	265.4	yes		
		Beer IIc	Beer IIc	235.7	yes		
			Beer IIc	221.7	yes		
			Beer IIc	270.2	yes		
Day 2	Beer I	Beer Ia	Beer Ia	282.0	yes	100%	
			Beer Ia	278.5	Yes		
			Beer Ia	270.2	Yes		
		Beer Ib	Beer Ib	248.3	Yes		
			Beer Ib	303.7	yes		
			Beer Ib	289.5	yes		
		Beer Ic	Beer Ic	301.9	yes		
			Beer Ic	271.5	yes		
			Beer Ic	226.6	yes		
	Beer II	Beer IIa	Beer IIa	288.0	yes		
			Beer IIa	232.3	yes		
			Beer IIa	279.6	yes		
		Beer IIb	Beer IIb	248.2	yes		
			Beer IIb	295.3	yes		
			Beer IIb	268.7	yes		
		Beer IIc	Beer IIc	208.9	yes		
			Beer IIc	283.3	yes		
			Beer IIc	229.4	yes		

**Table 3:** Composition and characteristics of beer samples.

	Number of Beers	Positive for Gluten	% (Positive/Number of Beer)	% (Positive/Total Beer Samples)
Total beer samples	107			
Yeast style				
Lager	49	10	20 (10/49)	9 (10/107)
Ale	46	16	35 (16/46)	15 (16/107)
Unknown	12	5	42 (5/12)	5 (5/107)
Label				
Low in gluten or with traces of gluten	12	4	33 (4/12)	4 (4/107)
Gluten-free	88	26	30 (26/88)	24 (26/107)
Unknown	7	1	14 (1/7)	1 (1/107)
Ingredients				
Contains cereals with gluten	70	24	34 (24/70)	22 (24/107)
Barley	59	16	27 (16/59)	15 (16/107)
Wheat	1	0	0 (0/1)	0 (0/107)
Oats	1	1	100 (1/1)	1 (1/107)
Barley and wheat	4	3	75 (3/4)	3 (3/107)
Barley and rye	1	0	0 (0/1)	0 (0/107)
Barley and oats	2	2	100 (2/2)	2 (2/107)
Wheat, barley, and rye	1	0	0 (0/1)	0 (0/107)
Wheat, barley, and oats	1	1	100 (1/1)	1 (1/107)
Unknown	15	6	40 (6/15)	6 (6/107)
Contains cereals with gluten and gluten-free	18	2	11.1 (2/18)	3 (3/107)
Barley and maize	7	1	14 (1/7)	1 (1/107)
Barley and millet	2	0	0 (0/2)	0 (0/107)
Wheat and millet	1	0	0 (0/1)	0 (0/107)
Barley, wheat, and quinoa	1	0	0 (0/1)	0 (0/107)
Barley and rice	3	0	0 (0/3)	0 (0/107)
Wheat, barley, rye, and rice	1	0	0 (0/1)	0 (0/107)
Barley, oats, and maize	1	0	0 (0/1)	0 (0/107)
Barley, rice, and maize	1	0	0 (0/1)	0 (0/107)
Barley, rice, and quinoa	1	1	100 (1/1)	1 (1/107)
	1			
Contains only gluten-free cereals	5	2	40 (2/5)	2 (2/107)
Maize	2	0	0 (0/2)	0 (0/107)
Millet	2	1	50 (1/2)	1 (1/107)
Buckwheat and rice	1	1	100 (1/1)	1 (1/107)
Country of origin				
Argentina	1	0	0 (0/1)	0 (0/107)
Belgium	10	1	10 (1/10)	1 (1/107)
Czech Republic	1	0	0 (0/1)	0 (0/107)
England	1	0	0 (0/1)	0 (0/107)
Finland	15	8	53 (8/15)	7 (8/107)
France	3	1	33 (1/3)	1 (1/107)
Germany	3	1	33 (1/3)	1 (1/107)
Italy	5	0	0 (0/5)	0 (0/107)
Luxembourg	2	2	100 (2/2)	2 (2/107)
Mexico	3	0	0 (0/3)	0 (0/107)
Poland	4	0	0 (0/4)	0 (0/107)
Portugal	1	0	0 (0/1)	0 (0/107)
Scotland	2	1	50 (1/2)	1 (1/107)
Spain	43	16	37 (16/43)	15 (16/107)
Switzerland	13	1	8 (1/13)	8 (1/107)

## Data Availability

Data is contained within the article or Supplementary Material.

## Supplementary Materials

The following supporting information can be downloaded at: https://www.mdpi.com/article/10.3390/foods12010160/s1, Table S1: Sensitivity G12/A1 LFIA for PTW-Cubus. LFIA, lateral flow immunochromatographic assays; Table S2: Peak area results of beer samples (gluten < LOD) obtained using the strip reader.
