# Supplementary material for: A Highly Sensitive Method for the Detection of Hydrolyzed Gluten in Beer Samples Using LFIA
Source: Foods. 2022 Dec 28;12(1):160. doi: 10.3390/foods12010160 (PMC9818069; doi:10.3390/foods12010160)
Supplement: Supplementary file 1 [file foods-12-00160-s001.zip › foods-2075218-supplementary.pdf]

Table S1. Sensitivity G12/A1 LFIA for PTW-Cubus. LFIA, lateral flow immunochromatographic assays.

| | ng/mL PTW-Cubus | | | | | | | | |
|---|---|---|---|---|---|---|---|---|---|
| | **0** | **1.25** | **2.5** | **5** | **10** | **15** | **20** | **25** | **Sensitivity** |
| **Analysis I** | Negative | Negative | Negative | **Positive** | **Positive** | **Positive** | **Positive** | **Positive** | |
| **Analysis II** | Negative | Negative | **Positive** | **Positive** | **Positive** | **Positive** | **Positive** | **Positive** | 5 ng/mL |
| **Analysis III** | Negative | Negative | Negative | **Positive** | **Positive** | **Positive** | **Positive** | **Positive** | |

Table S2. Peak area results of beer samples (gluten <LOD) obtained using the strip reader.

| Beer samples | Sample control number | Peak area |
|---|---|---|
| Beer I | 1 | 27,6 |
| | 2 | 23,5 |
| | 3 | 0 |
| | 4 | 0 |
| | 5 | 49,7 |
| Beer II | 6 | 0 |
| | 7 | 0 |
| | 8 | 39,4 |
| | 9 | 0 |
| | 10 | 0 |
| Beer III | 11 | 0 |
| | 12 | 43,6 |
| | 13 | 0 |
| | 14 | 0 |
| | 15 | 0 |
| Beer IV | 16 | 0 |
| | 17 | 0 |
| | 18 | 0 |
| | 19 | 0 |
| | 20 | 41,6 |
| Beer VI | 21 | 0 |
| | 22 | 31,6 |
| | 23 | 0 |

| | | |
|---|---|---|
| | 24 | 0 |
| | 25 | 24 |
